# Resveratrol Inhibits Hepatocellular Carcinoma Progression through Regulating Exosome Secretion

**DOI:** 10.2174/0929867331666230914090053

**Published:** 2024-02-26

**Authors:** Kun Tong, Pingfeng Wang, Ying Li, Yaoyao Tong, Xuejie Li, Shirong Yan, Pei Hu

**Affiliations:** 1 Department of Laboratory Medicine, Taihe Hospital, Hubei University of Medicine, Shiyan, China;; 2 Department of Laboratory Medicine, Huang Gang Central Hospital, Huanggang, China;; 3 Hubei Key Laboratory of Embryonic Stem Cell Research, Hubei University of Medicine, Shiyan, China;; 4 Institute of Biomedical Research, Hubei Clinical Research Center for Precise Diagnosis and Treatment of HCC, Taihe Hospital, Hubei University of Medicine, Shiyan, China;; 5 Department of Laboratory Medicine, General Hospital of the Yangtze River Shipping, Wuhan, China;; 6 Hubei Key Laboratory of Wudang Local Chinese Medicine Research, School of Pharmaceutical Sciences, Hubei University of Medicine, Shiyan, China

**Keywords:** Hepatocellular carcinoma, resveratrol, exosome, autophagy, lncRNA, nano-particle

## Abstract

**Background and Objectives:**

Resveratrol is a promising drug for tumor therapy, but its anti-tumor mechanism remains unclarified. The present study aimed to explore the effect of resveratrol on the secretion of exosomes and the role of resveratrol-induced exosomes in the progression of hepatocellular carcinoma.

**Methods:**

The number and contents of exosomes induced by resveratrol were determined by nanoparticle tracking analysis and high-throughput sequencing in Huh7 cells, respectively. Expression of Rab27a was assessed by Western blotting and immunofluorescence. Cell proliferation, migration and epithelial-mesenchymal transition were examined with the stimuli of resveratrol and exosomes, the activity of autophagy and wnt/β-catenin signaling induced by resveratrol-induced exosomes and knockdown of lncRNA SNHG29 were monitored by Western blotting and immunofluorescence.

**Results:**

It was found that resveratrol might inhibit the exosome secretion by down-regulating the expression of Rab27a, thereby suppressing the proliferation, migration and epithelial-mesenchymal transition of Huh7 cells. Moreover, resveratrol-induced exosomes could also inhibit the malignant phenotype of Huh7 cells *via* inhibiting the nuclear translocation of β-catenin and the activation of autophagy, which lncRNA SNHG29 might mediate.

**Conclusion:**

Resveratrol inhibits hepatocellular carcinoma progression by regulating exosome secretion and contents.

## 1 INTRODUCTION

Liver cancer ranks 6^th^ in the global incidence and is the second major cause of cancer-related death worldwide. Hepatocellular carcinoma (HCC), with high morbidity and mortality rates, accounts for about 70-90% of primary liver cancer, seriously threatening human health [1]. Nonalcoholic fatty liver disease (NAFLD) is the main cause of HCC in Westernized countries, and there is an unmet need to cure NAFLD to avoid its serious complications, such as liver cancer, by using natural products [2]. Mid-advanced liver cancer is still primarily treated with drugs, but the existing drugs have such deficiencies as drug resistance and side effects. Resveratrol, characterized by a wide range of biological activities, has displayed broad prospects in tumor therapy. As shown in multiple studies, resveratrol, through regulating the expression of a variety of tumor-related genes and participating in the cell proliferation, cell cycle, apoptosis and inflammation-related pathways [3-5], can restrain the progression of HCC. However, the specific anti-HCC mechanism of resveratrol still remains to be elucidated.

According to the latest studies, exosomes have tight associations with the occurrence and development of tumors [6]. However, there have been no reports yet on the effect of resveratrol on the secretion of exosomes in HCC. This study found that resveratrol could inhibit the exosome secretion through down-regulating Rab27a in Huh7 cells, and blocking exosome secretion could suppress the proliferation, migration and epithelial-mesenchymal transition (EMT) of Huh7 cells. In addition, resveratrol could induce composition changes in exosomes, increase the expression of long non-coding RNA(lncRNA)SNHG29 and induce its secretion by exosomes, thus restraining the malignant phenotype of Huh7 cells by inhibiting the Wnt/β-catenin pathway and autophagy activation [7, 8].

## 2 MATERIALS AND METHODS

### 2.1 Materials and Reagents

Resveratrol was purchased from Macklin (Shanghai, China), Dulbecco's modified Eagle medium (DMEM), fetal bovine serum (FBS), phosphate buffered saline (PBS), 0.25% trypsin and penicillin-streptomycin were purchased from Gibco (GrandIsland, NY, USA), RIPA lysis buffer, methyl thiazolyl tetrazolium (MTT), BCA protein assay kits were purchased from Beyotime Institute of Biotechnology (Shanghai, China), β-actin and Beclin1 monoclonal antibodies were purchased from Santa Cruz Biotechnology (CA, USA), proliferating cell nuclear antigen (PCNA), cyclinD1, E-cadherin, N-cadherin, Vimentin, β-catenin, c-Myc, glycogen synthase kinase-3β (GSK-3β), p62 and LC3 were bought from CST (Danvers, MA, USA), Histone-H3 was bought from Wuhan Proteintech Group, Inc. (Wuhan, China), electrochemiluminescence (ECL) solution and polyvinylidene fluoride (PVDF) membrane were bought from Millipore (Billerica, MA, USA), 4',6-diamidino-2-phenylindole (DAPI) was bought from Google Biological Technology Co., Ltd. (Wuhan, China), and PHK67 dye was bought from Umibio Co., Ltd (Shanghai, China).

### 2.2 Cell Culture

Huh7 cell lines purchased from the American Type Culture Collection (ATCC, Maryland, USA) were cultured in DMEM with 10% FBS and 5% CO_2_ at 37°C and digested with 0.25% trypsin, followed by passage.

### 2.3 MTT Assay

The Huh7 cells were inoculated into a 96-well plate (2×10^4^/well), with 5 replicates in each group. After being stimulated with the resveratrol or exosome, the cells were cultured with 5 mg/mL MTT for 4 h, added with 150 μL of dimethyl sulfoxide, and shaken at room temperature for 10 min. Finally, the absorbance was measured at a wavelength of 490 nm.

### 2.4 Wound Healing Assay

Upon reaching 80% confluence, the Huh7 cel ls were scratched, washed 3 times with PBS, cultured in DMEM containing different stimuli and photographed under an inverted microscope (Leica, Germany) at 0 h and 24 h. The area of the scratch region was calculated by ImageJ, and the wound healing rate was calculated.

### 2.5 Western Blotting

Cell lysates were harvested using RIPA lysis buffer. BCA protein assay kits were used to measure protein concentration. Then, the lysed proteins were separated by SDS-PAGE, transferred onto a PVDF membrane and blocked with 5% skim milk, followed by incubation with primary antibodies at 4°C overnight and with horseradish peroxidase (HRP)-conjugated anti-rabbit or anti-mouse secondary antibodies at room temperature for 2 h. Except for β-actin (1:5000), all the primary antibodies were diluted by 1:1000. Finally, the protein blots were developed using ECL solution and observed on a gel imaging analyzer.

### 2.6 Real-time Quantitative Polymerase Chain Reaction (qPCR)

The total RNA was extracted with TRIzol and reversely transcribed into cDNA using PrimeScript^TM^ RT reagent Kit with gDNA Eraser, followed by PCR using PowerUp^TM^ SYBR^TM^ Green Master Mix. The PCR primers are GAPDH-F: 5'-GGAGCGAGATCCCTCCAAAAT-3', GAPDH-R: 5'-GGCTGTTGTCATAC- TTCTCATG-3'. LncRNA-SNHG29-F: 5'-CTCCTGGGCTCAGATGGTCCTC-3', lncRNA-SNHG29-R: 5'-TTGTTGGGCATGATGGCAAGGG-3'. The reaction conditions are as follows: reverse transcription at 50°C for 2 min, pre-denaturation at 95°C for 2 min, and 40 cycles of denaturation at 95°C for 15 s and extension at 60°C for 1 min.

### 2.7 Immunofluorescence and Laser Confocal Microscopy

The Huh7 cells were placed on glass slides, subjected to the corresponding treatment, and then fixed with 4% paraformaldehyde, permeabilized with 0.1% Triton X-100, and blocked with 5% FBS, followed by incubation with primary antibody (1:200) overnight. Then, the cells were incubated with Alexa Fluor^®^ 488-labeled goat anti-rabbit or anti-mouse IgG (H+L) antibody at room temperature for 2 h, and the nuclei were stained with DAPI. Finally, the fluorescence intensity was detected by high-resolution laser confocal fluorescence microscopy.

### 2.8 Cell Transfection

mCherry-GFP-LC3 plasmids were purchased from Genomeditech, and siRNA targeting lncRNA SNHG29 was synthesized by Guangzhou RiboBio Co., Ltd. The Huh7 cells were inoculated into a 6-well plate and transfected with the plasmids or 50 nM siRNA using Lipofectamine 3000^TM^ following the instructions.

### 2.9 Autophagic Flux Assay

The cells were inoculated into a confocal dish and transfected with the mCherry-GFP-LC3 plasmids upon reaching about 70% confluence. 6 h later, the old medium was replaced with the exosome-containing medium. The autophagic flux was detected by high-resolution laser confocal fluorescence microscopy.

### 2.10 Extraction of Exosomes

The exosomes were separated and purified from the medium by ultra-high-speed centrifugation. Specifically, the medium supernatant was collected and centrifuged at 300×g for 10 min. Then the supernatant was collected and centrifuged at 2000×g for 10 min, and the supernatant was collected again and centrifuged at 18,000×g for 30 min. After the supernatant collected was filtered with a 0.22 μM filter, it was centrifuged at 120,000×g for 90 min. Finally, the resulting exosomes were resuspended with PBS, and their total protein concentration was measured by BCA kits.

### 2.11 Exosome Absorption Assay

The exosomes were stained with evenly mixed PHK67 dye following the instructions, placed on ice for 10 min and centrifuged at 120,000×g for 2 h. After the supernatant was discarded, the precipitate was resuspended with PBS and incubated with the cells for 24 h, followed by nuclear staining with DAPI. After mounting, the internalization of exosomes was observed by high-resolution laser confocal fluorescence microscopy.

### 2.12 Nude Mouse Tumorigenicity Assay

The nude mice (4-week-old female BALB/c nude mice, 18-20 g) were purchased from SPF Biotechnology Co., Ltd. (Beijing) and fed in the SPF laboratory animal center. They were randomly divided into 3 groups, with 3 mice in each group. The exosomes were extracted from Huh7 cells in normal exosome and resveratrol-induced exosome groups, and the concentration was adjusted with PBS to 200 μg/mL. 1×10^6^ Huh7 cells suspended by the exosomes were subcutaneously injected to establish the nude mice xenograft model. PBS was injected in the control group. The mice were euthanized and sacrificed 1 month later. The expressions of PCNA, c-Myc, p62 and Vimentin were detected by immunohistochemistry. The laboratory animal experiments strictly complied with the Guide for the Care and Use of Laboratory Animals issued by the National Institutes of Health.

### 2.13 Immunohistochemistry

The fixed tumor tissues were embedded in paraffin, sliced, deparaffinized with xylene, and washed with gradient alcohol. After antigen retrieval with citrate buffer and blockage of endogenous peroxidase with 3% H_2_O_2_, the sections were blocked with goat serum and incubated with primary antibodies at 4°C overnight. After washing with PBS, reaction enhancer solution was dropwise added to incubate the sections, followed by incubation with HRP-labeled goat anti-mouse/rabbit IgG polymerase. Finally, the staining effect was observed following DAB development. After hematoxylin counterstaining, the sections were photographed under a microscope.

### 2.14 Statistical Analysis

All assays were repeated 3 times, and SPSS 25.0 software was used for statistical analysis. All measurement data were expressed as mean ± standard deviation (mean ± SD). The means were compared by *t*-test between two groups, and the data were compared by analysis of variance among groups. *p<*0.05 was considered statistically significant.

## 3 RESULTS

### 3.1 Resveratrol Inhibited Exosome Secretion in Huh7 Cells by Down-regulating Rab27a

**Fig. (1):**
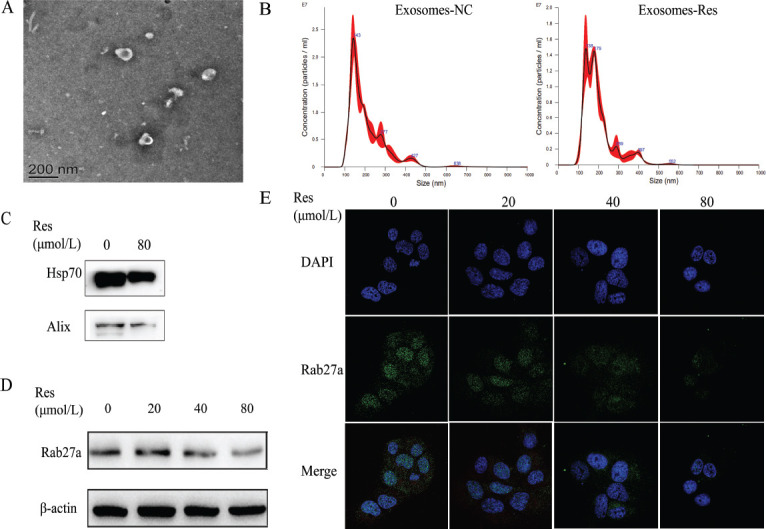
Resveratrol inhibited exosome secretion in Huh7 cells by down-regulating Rab27a. **(A)** The morphology of exosomes was identified by TEM. **(B)** The concentration of exosomes was determined by NTA. **(C)** The expression of exosomal protein markers (HSP70 and Alix1) in exosomes induced by resveratrol was detected by Western blot. **(D** and **E)** The expression of Rab27a induced by resveratrol was detected in Huh7 cells by Western blot and immunofluorescence, respectively. * *p<*0.05 *vs.* 0 μM. **p<0.01 *vs* 0 μM. Res: resveratrol.

To explore the effect of resveratrol on exosomes in HCC cells, the exosomes were extracted from Huh7 cells treated with 0 and 80 μmol/L resveratrol. It was observed under a transmission electron microscope (TEM) that vesicles were in a cup shape in both groups, and nanoparticle tracking analysis (NTA) showed that the diameter of 80% of the vesicles in both groups was 30-150 nm, conforming to the characteristics of exosomes (Figs. **1A** and **B**). Compared with that in the control group, the exosomes derived from Huh7 cells were significantly declined by 80 μmol/L resveratrol (Fig. **1C**), and the exosome marker proteins HSP70 and Alix were significantly down-regulated, suggesting that resveratrol inhibits the exosome secretion in Huh7 cells.

A previous study showed that the process where multivesicular bodies (MVBs) fusing with the plasma membrane are secreted outside of cells to form exosomes is regulated by Rab27a, and hence interfering with the expression of Rab27a can inhibit the exosome release. Western blotting results showed that after treating Huh7 cells with different concentrations of resveratrol for 24 h, the expression of Rab27a was down-regulated by resveratrol in a concentration-dependent manner (Figs. **1D** and **E**), suggesting that resveratrol suppresses the exosome secretion by down-regulating the expression of Rab27a in Huh7 cells.

### 3.2 Resveratrol Inhibited Proliferation, Migration and EMT of Huh7 Cells by Blocking Exosome Secretion

**Fig. (2):**
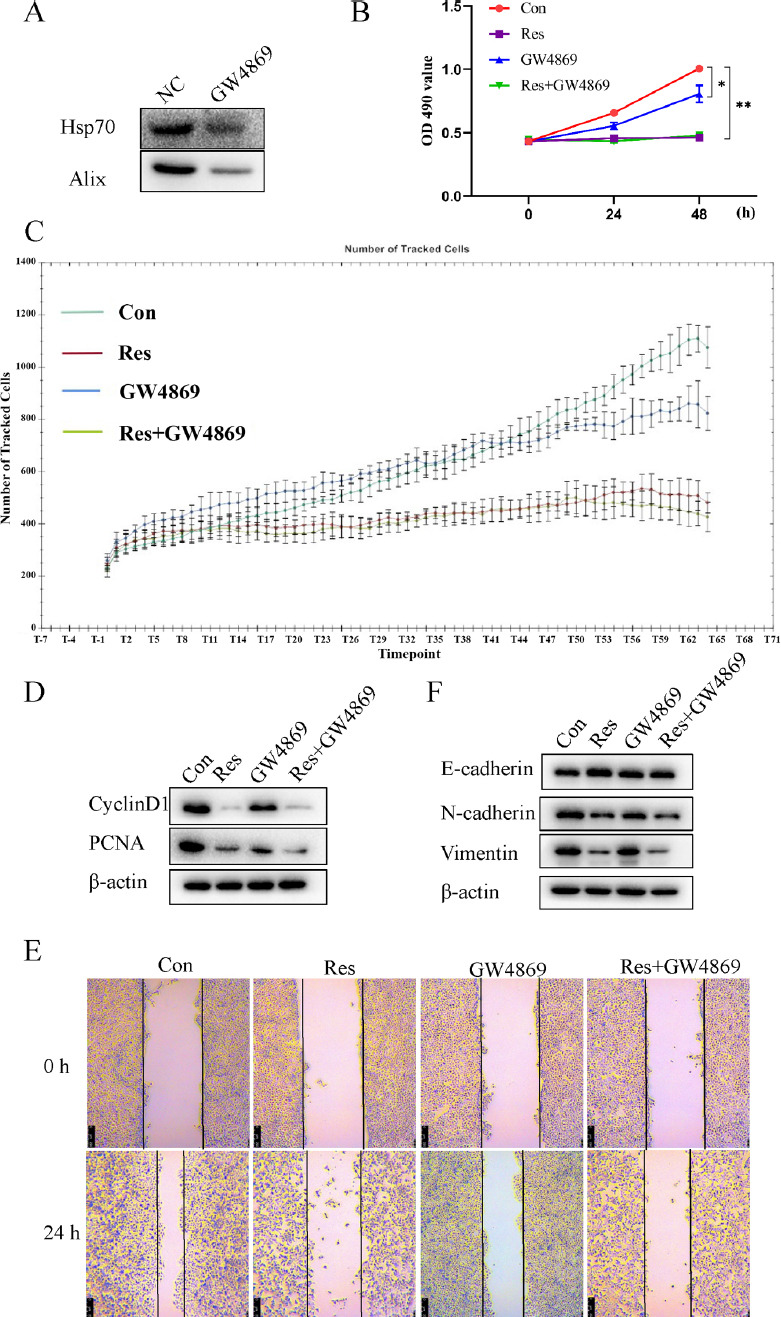
Resveratrol inhibited proliferation, migration and EMT of Huh7 cells by blocking exosome secretion. Huh7 cells were treated with 20 μmol/L GW4869 and/or 80 μmol/L resveratrol for 24 h, **(A)** The content of HSP70 and Alix1 in exosomes was detected by Western blot. **(B** and **C)** Cell viability and proliferation were monitored by MTT and high-content cell imaging analyzer, respectively. **(D)** The expression of cyclinD1 and PCNA was detected by Western blot. **(E)** Wound healing assay was performed to evaluate the cell migration. **(F)** The expression of EMT markers was detected by Western blot. * *p<*0.05 *vs.* Con. **p<0.01 *vs.* Con: control.

The ability of tumor-derived exosomes to facilitate malignant tumor progression has been demonstrated in numerous studies. To explore whether resveratrol restrains the progression of HCC by inhibiting exosome secretion, the exosome secretion inhibitor GW4869 was used as a control. First, it was verified that 20 μmol/L GW4869 could inhibit the exosome secretion in Huh7 cells (Fig. **2A**). Then the Huh7 cells were treated with 20 μmol/L GW4869 and/or 80 μmol/L resveratrol for 24 h. It was found that both GW4869 and resveratrol could significantly suppress the proliferation and migration of Huh7 cells, down-regulate the expressions of PCNA, cyclinD1, N-cadherin and Vimentin, and up-regulate the expression of E-cadherin (Figs. **2B**-**F**). However, the combination of the two showed a less significant synergistic effect. Based on the inhibitory effect of resveratrol on the exosome secretion in Huh7 cells, it is speculated that resveratrol may inhibit the proliferation, migration and EMT of Huh cells by blocking the exosome secretion.

### 3.3 Resveratrol Enhanced the Expression and Secretion of lncRNA SNHG29 in Huh7 Cells

**Fig. (3):**
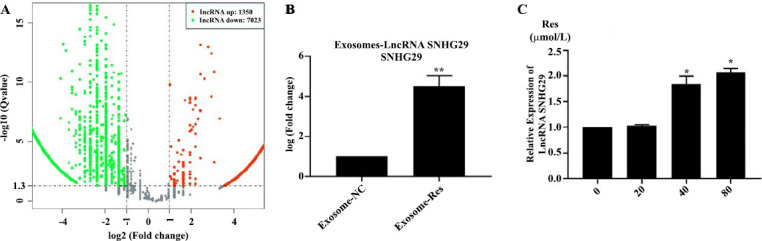
Resveratrol enhanced the expression and secretion of lncRNA SNHG29 in Huh7 cells. **(A)** High-throughput sequencing determined the differential expression of lncRNA induced by resveratrol. **(B)** The content of lncRNA SNHG29 induced by resveratrol in exosomes. **(C)** The expression of lncRNA SNHG29 induced by resveratrol in Huh7 cells.

As shown in many studies, resveratrol has a wide range of effects on the gene and protein expressions in tumor cells, so it is speculated that resveratrol can inhibit the exosome secretion in HCC and alter the exosome composition. Recently, the important role of lncRNAs in tumor progression has been proved. Therefore, high-throughput lncRNA sequencing was conducted on the composition of resveratrol-induced exosomes in Huh7 cells. 8373 differentially expressed lncRNAs were screened, including 1350 up-regulated ones and 7023 down-regulated ones. Of which, lncRNA SNHG29 was the most significantly up-regulated, and its expression in Huh7 cells could also be greatly up-regulated by 80 μmol/L resveratrol (Fig. **3**). It can be seen that resveratrol can up-regulate the expression of lncRNA SNHG29 and induce its secretion by exosomes in Huh7 cells.

### 3.4 Resveratrol-induced Exosomes Inhibited the Progression of HCC

As proved above, resveratrol can induce composition changes in exosomes in Huh7 cells. However, the effect of resveratrol-induced exosomes on the malignant phenotype of tumor cells remains to be further studied. Therefore, exosomes were extracted from normal cells (Exosome-NC) and resveratrol-stimulated cells (Exosome-Res). To confirm that exosomes can be internalized by Huh7 cells, the exosomes were labeled with PKH67. As shown in Fig. (**4A**), green fluorescence around the cells was observed under the high-resolution laser confocal fluorescence microscope, proving that the exosomes extracted *in vitro* can be absorbed by Huh7 cells.

**Fig. (4):**
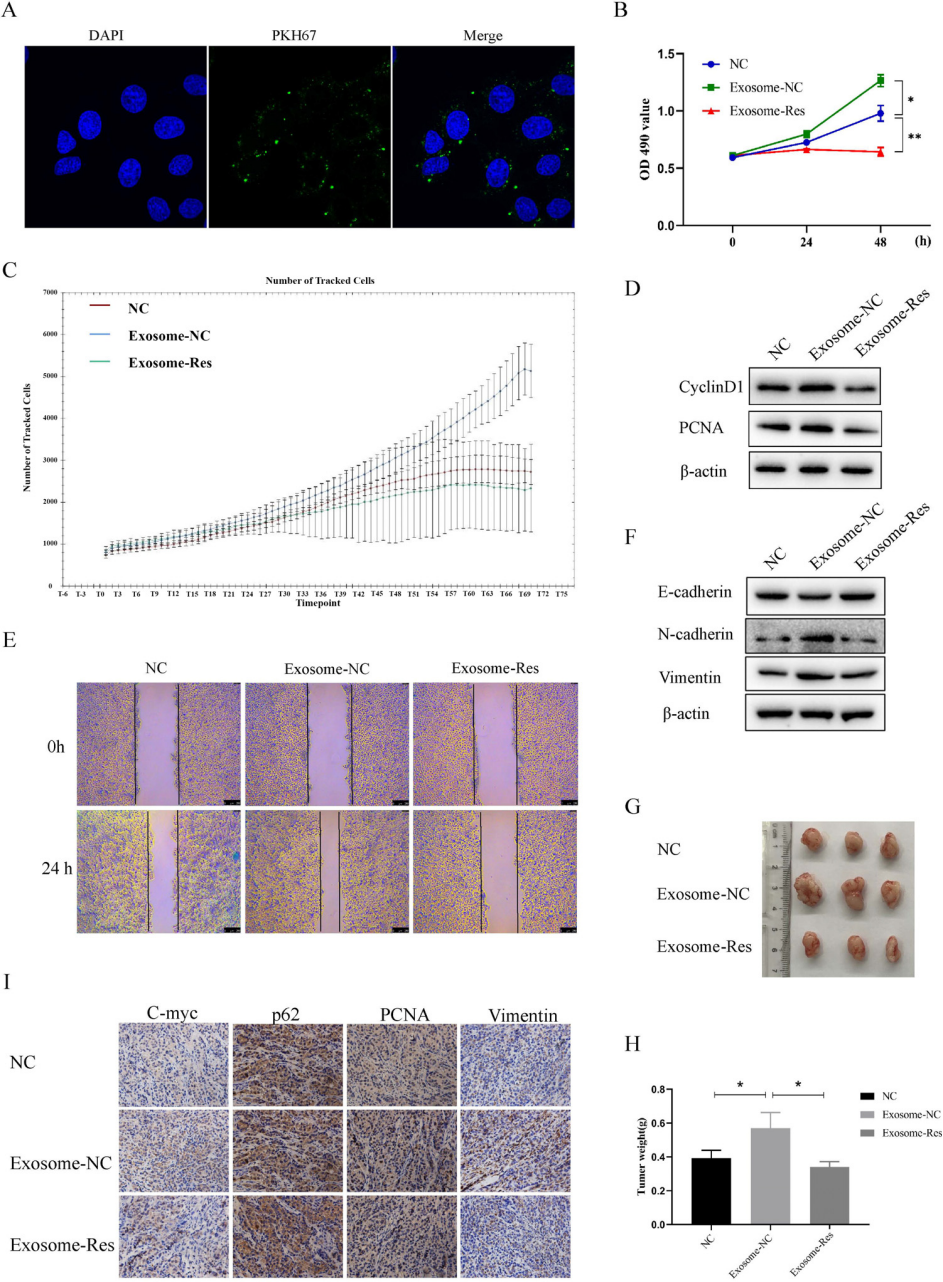
Resveratrol-induced exosomes inhibited the progression of HCC. **(A)** Absorption of exosomes stained by PKH was measured by high-resolution laser confocal fluorescence microscopy. **(B** and **C)** Cell viability and proliferation of Huh7 cells treated with Exosome-NC or Exosome-Res were measured by MTT and high-content cell imaging analyzer, respectively. **(D)** The expression of cyclinD1 and PCNA was detected by Western blot. **(E)** Wound healing assay was performed to evaluate the cell migration. **(F)** The expression of EMT markers was detected by Western blot. **(G)** The tumor extracted from the xenograft mice treated with different exosomes. **(H)** The tumor weight was measured in different groups. I, The expression of c-myc, p62, PCNA and vimentin in tumors were determined by immunohistochemistry.

Next, the cells were incubated with the extracted exosomes (20 µg/mL) while the same amount of PBS was added to the control group. Compared with controls, exosomes derived from the normal cells could greatly enhance the proliferation and migration of Huh7 cells, up-regulate the expressions of PCNA, cyclinD1, N-cadherin and Vimentin, and down-regulate the expression of E-cadherin, demonstrating that Huh7 cells-derived exosomes can facilitate proliferation, migration and EMT of Huh7 cells. At the same time, the malignant phenotypes were suppressed by Exosome-Res (Figs. **4B**-**F**). To sum up, Exosome-Res restrain the proliferation, migration and EMT of Huh7 cells.

The xenografts were established further to explore the effect of resveratrol-induced exosomes on tumor growth. As shown in Figs. (**4G** and **H**), the tumor size was significantly larger in the Exosome-NC group than in the control group. At the same time, it was significantly smaller in the Exosome-Res group, suggesting that Exosome-Res can suppress tumor growth in mice. Besides, it was found by immunohistochemistry that the expressions of PCNA, c-Myc and Vimentin significantly rose, while the expression of p62 significantly declined by Exosome-NC (Fig. **4I**). The opposite tendency was shown in the Exosome-Res group, consistent with the *in vitro* assay.

### 3.5 Resveratrol-induced Exosomes Inactivated Wnt/β-catenin Pathway and Autophagy of Huh7 Cells

**Fig. (5):**
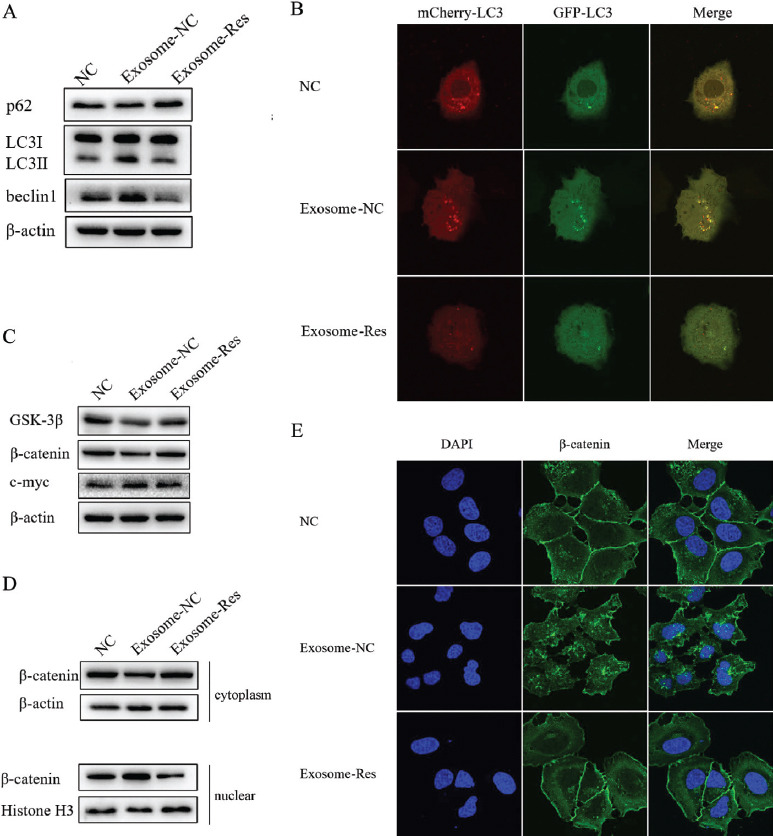
Resveratrol-induced exosomes inactivated Wnt/β-catenin pathway and autophagy of Huh7 cells. **(A)** The expression of autophagy markers induced by Exosome-NC or Exosome-Res for 24 h. **(B)** Autophagy flux induced by Exosome-NC or Exosome-Res was monitored by laser confocal fluorescence microscopy. **(C)** The expression of GSK3β, β-catenin, and c-myc induced by Exosome-NC or Exosome-Res for 24 h in Huh7 cells were determined. **(D** and **E)** The expression and cellular distribution of β-catenin in cytoplasm and nuclei induced by Exosome-NC or Exosome-Res were determined by Western blot and immunofluorescence.

It has been proved previously that resveratrol can induce autophagy of HCC cells. To explore whether autophagy is involved in the regulatory effect of Exosome-Res on the progression of HCC, Huh7 cells were re-incubated with the exosomes (20 µg/mL). As shown in Fig. (**5A**), Exosomes derived from Huh7 cells could up-regulate the autophagy marker protein Beclin1 expression, down-regulate the expression of p62, and facilitate the conversion of LC3-I to LC3-II. The increased autophagic flux could also be seen under the laser confocal microscope (Fig. **5B**). Compared with Exosome-NC, Exosome-Res inactivated the autophagy and blocked the autophagy flux.

As a regulator for tumor proliferation and metastasis, the Wnt/β-catenin signaling pathway becomes abnormal in the proliferation and migration of tumor cells. The present study has demonstrated that exosomes derived from Huh7 cells up-regulated the expression of c-Myc and down-regulated the expressions of GSK-3β and β-catenin (Fig. **5C**). At the same time, Exosome-Res reduced the expression of c-Myc, and increased the expressions of GSK-3β and β-catenin compared with Exosome-NC (Fig. **5C**), indicating that Exosome-Res can inhibit the Wnt/β-catenin signaling pathway. Furthermore, the intracellular localization of β-catenin was detected. The results revealed that the tumor exosomes lowered the content of β-catenin in the cytoplasm and raised the level of β-catenin in the nucleus, while Exosome-Res reversed it (Figs. **5D** and **E**). The above findings validate that Exosome-Res inhibit the activation of the Wnt/β-catenin signaling pathway by preventing β-catenin from entering the nucleus.

### 3.6 LncRNA SNHG29 Suppressed the Autophagy Activation and Nuclear Translocation of β-catenin in Huh7 Cells

**Fig. (6):**
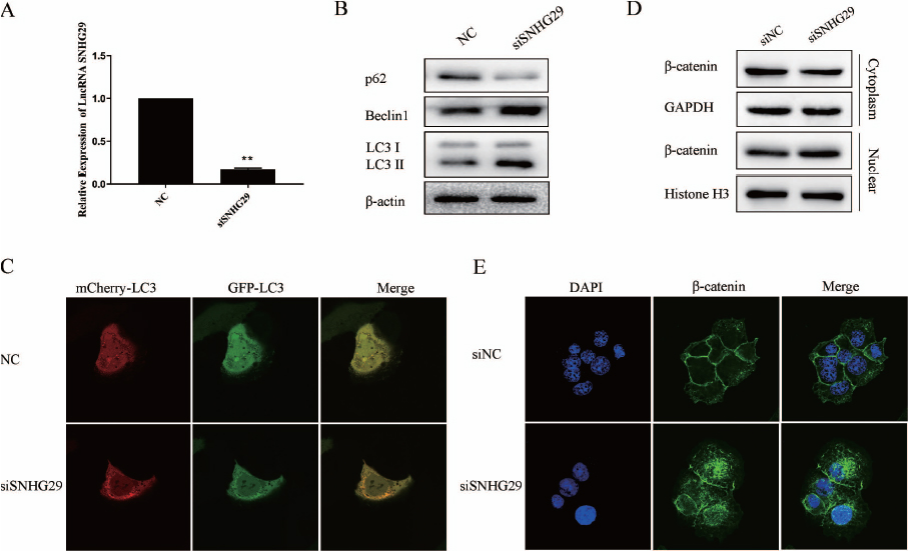
LncRNA SNHG29 suppressed the nuclear translocation of β-catenin and autophagy of Huh7 cells. **(A)** The expression of LncRNA SNHG29 in Huh7 cells transfected with si-SNHG29. **(B)** The expression of autophagy markers in Huh7 cells transfected with siSNHG29. **(C)** Autophagy flux was monitored by laser confocal fluorescence microscopy. **(D** and **E)** The expression and cellular distribution of β-catenin in cytoplasm and nuclei induced by siRNA targeting lncRNA SNHG29 were determined by Western blot and immunofluorescence. siNC: siRNA for negative control. siSNHG29: siRNA targeting lncRNA SNHG29.

It has been proved that resveratrol can induce the increased secretion of lncRNA SNHG29 in Huh7 cells, so are the regulatory effect of Exosome-Res on autophagy and nuclear translocation of β-catenin mediated by lncRNA SNHG29? The Huh7 cells were transfected with siRNA targeting lncRNA SNHG29 to answer this question. The results showed that after silencing of lncRNA SNHG29 (Figs. **6A**-**C**), the expression of Beclin1 was up-regulated, the expression of p62 was down-regulated, the conversion of LC3-I to LC3-II was enhanced, and increased autophagic flux could also be seen under the laser confocal microscope, indicating that lncRNA SNHG29 can inhibit the autophagy of Huh7 cells. Additionally, the knockdown of lncRNA SNHG29 decreased the content of β-catenin in the cytoplasm while increasing the level in the nucleus (Figs. **6D** and **E**). According to the above results, the inhibitory effect of resveratrol-induced exosomes on the autophagy and nuclear translocation of β-catenin of Huh7 cells may be mediated by lncRNA SNHG29.

## 4 DISCUSSION

Resveratrol is a kind of plant estrogen that can be obtained from food, and it possesses a wide range of pharmacological effects, including cardioprotective, antioxidant, anti-inflammatory and anti-tumor properties [9], with very weak toxic and side effects on normal somatic cells. Therefore, resveratrol has become a potential therapeutic drug for many diseases. Regarding its anti-tumor properties, resveratrol can restrain tumor development by regulating the expression of different tumor-related genes and the activity of signal transduction pathways. Although it has been widely studied in HCC, the mechanisms remain to be fully clarified.

Exosomes are vesicle-like bodies (about 30-150 nm in diameter) actively secreted by various viable cells to the outside of cells, which carry multiple biologically active cellular components such as proteins, DNAs, mRNAs and miRNAs. They are important players in the pathophysiological processes of the human body and are implicated in cellular waste management and intercellular communication [10]. Exosomes are derived from intraluminal vesicles (ILVs) in the endosomal compartment. Specifically, the plasma membrane is internalized by cells through endocytosis, and then endosomes are generated. Multiple endosomes fuse from early endosomes, which are further transformed into MVBs. After fusing with the plasma membrane, MVBs are released to the outside of cells, which are considered exosomes. Two small GTPases of the Rab family (Rab27a and Rab27b) were identified in regulating the docking and fusion between MVBs and the plasma membrane. It has been found that Rab27a regulates exosome secretion *in vitro* and *in vivo* [11], and its overexpression facilitates the invasion and migration of melanoma cells [12]. In this study, the results showed that resveratrol inhibited the exosome secretion in HCC cells and down-regulated the expression of Rab27a in a concentration-dependent manner, suggesting that resveratrol may suppress the exosome secretion in HCC cells through down-regulating the expression of Rab27a.

Recently, increasing studies have shown that exosomes have close associations with the occurrence and development of tumors, including tumor proliferation, migration, immune escape and angiogenesis [13-16]. The exosome secretion is stronger in tumor cells than in normal viable cells [17]. Suppressing exosome secretion is potentially a new therapeutic strategy for tumors [18]. Akihiro *et al*. found that GW4869 can inhibit the regulatory effects of mouse melanoma cell-derived exosomes on the proliferation and apoptosis of B16BL6 cells [19]. Similarly, the results of this study revealed that both resveratrol and GW4869 restrained the proliferation, migration and EMT of HCC cells. However, the combination of the two showed a less significant synergistic effect. Therefore, it is speculated that inhibiting exosome secretion may be another novel mechanism of resveratrol in suppressing the progression of HCC.

As an important carrier of intercellular communication, exosomes play important roles in tumors mainly through their biologically active components in the tumor microenvironment [20]. Exosomes can transfer oncogenic RNAs and proteins in HCC cells into adjacent ones, thus enhancing the migration and invasion of HCC cells [21, 22]. In addition, exosomes from highly metastatic HCC cells can be absorbed by lowly metastatic HCC cells, thereby promoting the migration and invasion of the latter [23]. This study found that resveratrol could induce the expression and secretion of lncRNA SNHG29, and exosomes induced by resveratrol can inhibit the proliferation, migration and EMT of Huh7 cells. It can be inferred that resveratrol can inhibit the exosome secretion in HCC and induce exosomes with anti-cancer activity to suppress the progression of HCC.

Furthermore, the present study revealed that autophagy and Wnt/β-catenin pathway were involved in the inhibitory effect of resveratrol-induced exosomes on the proliferation, migration and EMT of HCC cells. Autophagy, a conserved self-degradation system, is crucial for keeping cellular homeostasis under stress, which dynamically promotes or suppresses tumors in different environments and stages of tumor development [24]. It has become clear that autophagy is also implicated in the regulatory effect of exosomes on tumors [25, 26]. In this study, autophagy of HCC cells was induced by HCC-derived exosomes but inactivated by resveratrol-induced exosomes. In addition, wnt/β-catenin signaling pathway was also inhibited by resveratrol-induced exosomes through blocking β- catenin from entering the nucleus, and the further study revealed that lncRNA SNHG29 mediated the effects of resveratrol-induced exosomes on autophagy and wnt/β-catenin signaling.

## CONCLUSION

Through exploring the effect of resveratrol on the exosome secretion in HCC and the effect of resveratrol-induced exosomes on the HCC progression in this study, the anti-HCC mechanism of resveratrol is further clarified, and blocking the exosome secretion is considered a new therapeutic method for HCC. In addition, the possible important role of lncRNA SNHG29 in the progression of HCC was verified in this study. However, its effect on tumors has been rarely studied, so it is necessary to further investigate the role and mechanism of lncRNA SNHG29 in the progression of HCC.

## ETHICS APPROVAL AND CONSENT TO PARTICIPATE

The nude mouse tumorigenicity assay were approved by the Institutional Animal Care and Use Committee of Hubei University of Medicine (approval no. 2021-062).

## HUMAN AND ANIMAL RIGHTS

 The laboratory animal experiments strictly complied with the Guide for the Care and Use of Laboratory Animals issued by the National Institutes of Health. All methods are reported in accordance with the ARRIVE Guidelines.

## CONSENT FOR PUBLICATION

Not applicable.

## AVAILABILITY OF DATA AND MATERIALS

The data and supportive information are available within the article.

## FUNDING

The present study was supported by the Hubei Provincial Natural Science Foundation (2020CFB235), the Research Program for Hepatobiliary and Pancreatic Malignancy of Chen-Xiaoping Foundation (CXPJJH12000001-2020340).
